# Modeling homosynaptic and heterosynaptic plasticity with a single neuromemristive synapse

**DOI:** 10.1016/j.jare.2025.10.031

**Published:** 2025-10-22

**Authors:** Zubaer Ibna Mannan, Sami Azam, Ram Kaji Budhathoki, MD Nur Alam, Hyongsuk Kim

**Affiliations:** a Department of Computer Science and Engineering, East West University, Jahurul Islam Ave., Dhaka 1212, Bangladesh; b Faculty of Science and Technology, Charles Darwin University, NT 0810, Australia; c Department of Electrical and Electronics Engineering, School of Engineering, Kathmandu University, Dhulikhel, Kavre, 45200, Nepal; d Department of Artificial Intelligence, Kyungdong University Global, Geosong, Gangwon-do 46(4)-24764, the Republic of Korea; e Division of Electronics Engineering, and Intelligent Robot Research Center, Jeonbuk National University, Jeonju, Jollabuk-do 567-54896, the Republic of Korea

**Keywords:** Synapse, Long-term potentiation (LTP), Long-term depression (LTD), Short-term facilitation (STF), Short-term depression (STD), Homeostatic, Modular input-specific, Associativity, Neuromorphic circuit, Memristor and memristive system, Artificial synapse

## Abstract

**Introduction:**

Efforts are being made to design a brain-like intelligence due to its robustness, synaptic modification (i.e., learning and memory), analog synaptic multiplication, multi-state storage, ultra-low power consumption, and parallel computation. However, current bioelectronic and biomedical technologies have yet to fully replicate brain-like intelligence. In particular, devices that can emulate both homosynaptic and heterosynaptic plasticity remain extremely limited.

**Objectives:**

This work presents a neuromemristive synapse capable of replicating key biological features, including homosynaptic and heterosynaptic plasticity. The proposed synapse uses a memristor, a promising candidate for achieving bio-realistic features of synapse due to its low power consumption, multi-state operation, analog behavior, high data storage durability, and CMOS compatibility.

**Methods:**

The artificial synapse is designed as a composite 1-port structure consisting of a memristor (M) and a controlled capacitor (CCon). The memristor is responsible for emulating synaptic plasticity in response to distinct brainwave patterns, while the capacitor modulates the discharge rate through the memristor. During the active input phase (synaptic potentiation), the composite 1-port charges up. During the inactive phase (synaptic depression), CCon governs the discharging of the memristor, enabling full or partial discharging through memory fading or homosynaptic and heterosynaptic depression pathways.

**Results:**

We designed the proposed synapse in SPICE and validated its bio-functionalities through various simulations. The proposed synapse demonstrates low power consumption and replicates key neurobiological processes for learning and memory such as heterosynaptic homeostasis, modular input specificity, associativity, and homosynaptic long-term and short-term potentiation and depression (LTP, LTD, STF, STD), along with memory fading effect (MFE), and strong stimulation (SST).

**Conclusion:**

The proposed synapse bio-realistically mimics synaptic plasticity, neurotransmitter dynamics, and neuronal responses. Implemented using off-the-shelf components, it supports both volatile and non-volatile modes, making it suitable for CMOS integration. This enables advancements in spiking neural networks, brain function analysis, and scalable neuromorphic computing systems.

## Highlights

• An analog neuromemristive synapse is designed to mimic the bio-realistic characteristics of neurons, synapses, and synaptic plasticity. The artificial synapse uses a single memristor to support homo- and hetero- synaptic long-term and short-term plasticity.
• It imitates the homeostatic, modular input specificity, and associativity roles of the heterosynaptic plasticity. It also mimics homosynaptic short-term facilitation (STF) and depression (STD), long-term potentiation (LTP) and depression (LTD), as well as reuptake processes and strong stimulation phenomena.
• This artificial synapse can be served as a potential substitute for in-vivo and in-vitro analyses in academia. In addition, the proposed synapse functions in both volatile and nonvolatile modes, evincing it suitable for applications in Spiking Neural Networks (SNNs) to support Hebbian, anti-Hebbian, and associative learning paradigms.
• Particularly, it overcomes the limitations inherent in graphene, liquid crystal carbon nanotube composites, subthreshold analog and transistor-based CMOS neural circuits, and traditional Von Neumann architectures.
• The power- and area-efficient artificial neuromemristive synapse can be ported in miniature CMOS ICs, which paves the way to build artificial neuronal or neural networks that exhibit human brain-like intelligence.

## Graphical abstract

A neuromemristive synapse is developed that can emulate both homo- and heterosynaptic plasticity, showcasing the homeostatic and modular input-dependent roles of heterosynaptic plasticity, along with homosynaptic long-term and short-term potentiation and depression, and functioning effectively within brainwave frequency ranges.

**Figure:**
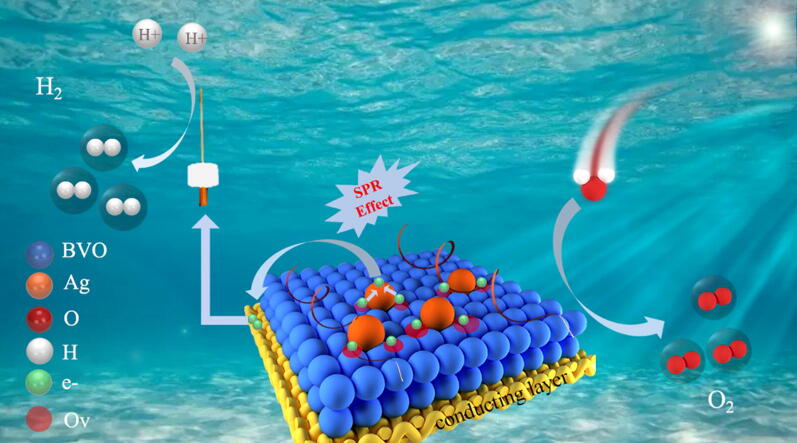

## Introduction

Electronic circuits that mimic biological mechanisms are incredibly efficient, robust, and have enormous parallel processing capabilities [1]. The development of a brain-like intelligence is currently achievable because of recent developments in bioelectronics and neuromorphic engineering [2,3]. These bio-inspired neuromorphic circuits are considered to be the next generation of computing platforms. They surpass traditional Von-Neumann architectures because of their high parallel processing, low power consumption, and high adaptability [4]**.** Recent advancements in nano-scale CMOS technologies have made it possible for researchers to develop large-scale neuromorphic circuits using very large-scale integration (VLSI) hardware. In recent years, researchers have used different technologies to develop neuromorphic devices that include CMOS [5,6], Subthreshold CMOS [7], OxRAM [8], Switched-Capacitor (SC) [9], Spintronic [10], Graphene Transistor [11], Barristor [12], Liquid Crystal Carbon Nanotube (LC-CNT) Composites [13], and Memristor [[14], [15], [16], [17], [18], [19], [20], [21], [22], [23], [24], [25]]. Among all of these elements, memristor is considered one of the most preeminent elements for designing neuromorphic integrated circuits because it works like a biosynapse and has low energy consumption, multiple-state operation, great scalability, higher durability for storing data, and CMOS compatibility [15,26,27]. Moreover, CMOS is energy inefficiency in analog synaptic operation [28], Subthreshold CMOS suffers with low operating speed [29], OxRAM requires high-voltage electroforming to initialize the switching medium [30], and Switched-Capacitor (SC) is prone to leakage and device variation [31]. In addition, Spintronic Devices exhibits stochastic switching [32], Graphene Transistor lacks of a band gap (i.e., zero bandgap) which makes it unsuitable for low-power neuromorphic circuits [33], Barristor suffers with manufacturing scalability, variability, and integration challenges [34], and LC-CNT Composites relies on physical alignment-assisted switching, which suffers from mechanical instability and inconsistent behavior under scaling [13]. Therefore, numerous research activities are focused on neuromorphic computation using the memristive system.

In this paper, an artificial biosynapse was developed using a composite 1-port of a memristor (M) and a controlled capacitor (C_Con_). The proposed neuromemristive synapse impersonates the physiological acts of homo and hetero synaptic plasticity in accordance with the distinct brain states thereby correlating to a particular frequency of the brainwaves. The memristance and voltage across the memristor represent the artificial synaptic strength and synaptic voltage, respectively. The C_Con_ capacitor controls the rate of discharging through the memristor (M). During homo or hetero synaptic potentiation, the composite 1-port is charging in the active cycle of homo or hetero synaptic input stimuli. However, during the inactive cycle of stimulations, the C_Con_ capacitor forces the memristor to either fully or partially discharge through M. Similar to biological synapses, the artificial synapse distinguishes between homosynaptic short-term (STF and STD) and long-term (LTP and LTD) plasticity, shown in Table. 1, based on repetition time of the input cycle. The proposed synapse also features separate pathways through M and C_Con_ to facilitate the different synaptic weakening rates for homo and hetero synaptic depressions and reuptake processes.

**Table 1:** Abbreviations of the Synaptic Terms.

Terms	Abbreviations	Terms	Abbreviations
Long-Term Potentiation	LTP	Non-plastic Synaptic Response	NSR
Long-Term Depression	LTD	Strong Stimulation	SST
Short-Term Facilitation	STF	Heterosynaptic Facilitation	HTE_FAC
Short-Term Depression	STD	Heterosynaptic Depression	HTE_DEP
Memory Fading Effect	MFE	Memristive Synaptic Efficacy	M_Syn_

The proposed neuromemristive synapse is beneficial than existing state-of-the-art artificial neuromorphic synapses in a number of ways. Neuromorphic excitatory synapses shown in [5] need twice as much hardware as compared to our proposed synapse in order to support only STDP (spike-timing-dependent plasticity). The self-sufficient proposed model is more compatible and uses less space than the digitally controlled neuromorphic circuit in [6], which needs more circuits to implement synaptic plasticity. The sub-threshold CMOS iono-neuromorphic model in [7] requires nonvolatile digital storage to store synaptic modification and is unable to exhibit short-term synaptic plasticity compared to our proposed synapse. To imitate the STP and LTP phenomena, the proposed neuro-memristive synapse requires only a single memristor, unlike the OxRAM synapse in [8], which requires 10 and 20 OxRAM devices, respectively. Moreover, the proposed synapse is bio-realistically more effective, energy-efficient, and area-efficient than the switched-capacitor-based conductive synapse in [9], which requires multiple switches, a couple of capacitors, and an amplifier to implement a single synaptic conductance. Both the hybrid spintronic-CMOS synapse in [10], the twisted bilayer graphene transistor synapse in [11], and the CMOS-memristive synapse in [19] exhibit only STDP mechanisms. The synaptic barristor in [12] exhibits LTP, LTD, STP, and STD plasticity but doesn’t exhibit heterosynaptic plasticity. The liquid crystal-carbon nanotube (LC-CNT) synapse in [13] and the neuro-transistor in [20] are unable to exhibit homo and hetero synaptic depressions, whereas the solid-state PEN-based memristive synapse in [18] only demonstrates heterosynaptic plasticity. The memristive synapses in [[21], [22], [23]] are unable to exhibit heterosynaptic plasticity. In contrast to these synapses, the proposed neuro-memristive synapse demonstrates both homo and hetero synaptic facilitation and depression, as well as memory-fading effects. Unlike the mathematical [16] and macro [17] models of the memristive synapse, our proposed synapse is implemented in a circuit with off-the-shelf components. In addition, the proposed neuro-memristive synapse can operate at different frequency range and pulse width (1 ∼ 3 ms) of the brainwaves. In contrast, the WSe_2_-based memtransistor in [24] and LiNbO_3_ memristor in [25], which required 3 V ∼ 20 V and ±4 V pulse amplitude, respectively, with a 50 ms pulse width.

The main contribution of this paper is the design of an analog neuromemristive synapse that mimics bio-realistic characteristics of neurons, synapses, and synaptic plasticity. The artificial synapse uses a single memristor to support homo- and hetero- synaptic long-term and short-term plasticity. It imitates the homeostatic, modular input specificity, and associativity roles of the heterosynaptic plasticity. It also mimics homosynaptic short-term facilitation (STF) and depression (STD), long-term potentiation (LTP) and depression (LTD), as well as reuptake processes and strong stimulation phenomena. This artificial synapse can be served as a potential substitute for in-vivo and in-vitro analyses in academia. In addition, the proposed synapse operates in both volatile and nonvolatile modes. This makes it suitable for Spiking Neural Networks (SNNs), where it can support Hebbian, anti-Hebbian, and associative learning paradigms. Particularly, it overcomes the limitations of graphene, liquid crystal carbon nanotube composites, subthreshold analog and transistor-based CMOS neural circuits, and traditional Von Neumann architectures. This advantage allows it to be integrated into miniature CMOS ICs, paving the way for artificial neural networks that exhibit human brain-like intelligence.

The rest of the paper is organized as follows: Section 2 describes the bio-synapse and its plasticity, and Section 3 discusses the operation of the proposed neuro-memristive synapse. Section 4 presents the simulations and results, followed by concluding remarks in Section 5.

## Synapse, plasticity and brainwaves

**Fig. 1:**
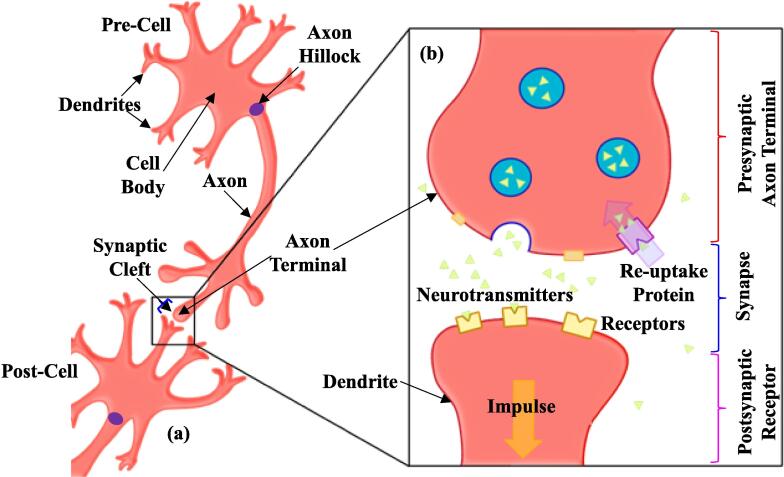
Schematic illustration of neuron–neuron communication and synaptic structure. (a) It depicts the primary components of a neuron, including the cell body, dendrites, axon, and axon terminal. (b) It provides a detailed cross-section of a synapse, illustrating the process of synaptic transmission, neurotransmitter release, receptor binding, and reuptake.

Neurons or nerve cells are the building blocks of the nervous system that transmit information electrical-chemically via synapse. Synapse, a junction between two neurons, plays a crucial role in transmitting signals between neurons, allowing for the integration of information and the coordination of various brain functions. Synapse allows the release of neurotransmitters (such as acetylcholine, dopamine, serotonin, glutamate, and so on) from the presynaptic neuron into the synaptic cleft,^1^ shown in Fig. 1(a), where they bind to receptors on the postsynaptic neuron, shown in Fig. 1(b), initiating a series of opening and closing of ion channels [35,36]. The release of different types of neurotransmitters have an impact on the ion channel behavior and the outcomes of postsynaptic cells. For instance, excitatory neurotransmitters such as acetylcholine or glutamate depolarize postsynaptic cells, increasing the probability of postsynaptic firings, a phenomenon known as synaptic potentiation, while inhibitory neurotransmitters such as GABA or dopamine suppress post-firings by hyperpolarizing the postsynaptic cell, leading to synaptic depression [37].When the postsynaptic neuron depolarizes, it membrane potential increases from resting membrane potential (typically −70 mV) and when it reaches the threshold (typically −55 mV), it generates an all-or-none electrical impulse, namely action potential (AP). Generation of such action potentials in synaptic transmission is proportional to the probability and pattern of neurotransmitter diffusion from presynaptic neuron and the receptor sensitization of postsynaptic neuron. The continual diffusion of neurotransmitters increases the synaptic efficacy or strength of the synapse between two neurons [38,39]. However, synapse utilizes the reuptake process to remove the neurotransmitters from the synaptic cleft to regulate the strength of synapse or to terminate the synaptic transmission of signals between neurons. Reuptake process, shown in Fig. 1(b), absorbs the diffused neurotransmitters from synaptic cleft through neurotransmitter transporters and brings back to presynaptic neurons. The reuptake process plays a crucial role in regulating the concentration of neurotransmitters in the synaptic cleft, thereby influencing the strength (*i.e.*, decreasing the synaptic strength) and duration of synaptic transmission [37]. The physiological attributes of synaptic depression caused by reuptake and inhibitory neurotransmitters should be addressed separately. This is because inhibitory neurotransmitters hyperpolarize the post-cell, which results in post-membrane potential being reduced to −110 mV ∼ −150 mV, whereas in the reuptake process, post-membrane potential remains same as resting potential. The reuptake process can be related to the memory fading effect (MFE) in an electronic circuit [21,22].

**Fig. 2:**
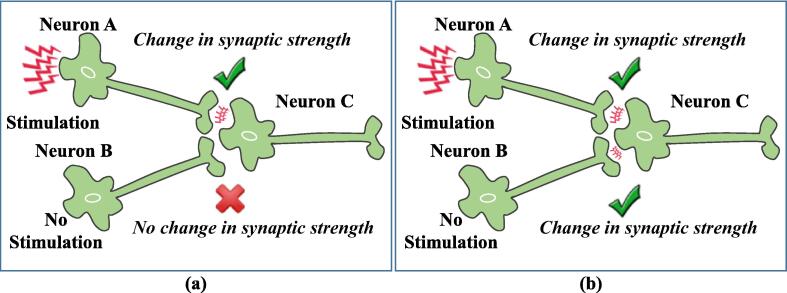
(a) Illustration of homosynaptic plasticity where concurrent firing in Neuron A only induces changes in the synaptic connection from Neuron A to Neuron C. (b) A model of heterosynaptic plasticity, where concurrent firing in Neuron A induces a change in the synaptic connection from Neuron B to Neuron C.

Synaptic plasticity is the biological mechanism by which specific patterns of synaptic activities lead to changes in synaptic strength or efficacy. It is the ability of synapses to increase or decrease its efficacy and is thought to be one of the most important neurochemical foundations of learning and memory [37]. Depending on the input specificity, synaptic plasticity of a synapse is characterized in two types: homosynaptic plasticity and heterosynaptic plasticity, as shown in Fig. 2. Homosynaptic plasticity is input-specific or associative, *i. e.*, synaptic efficacy changes only at postsynaptic targets specifically stimulated by a presynaptic neuron. It is a form of synaptic plasticity where changes occur specifically at the synapses that are activated, as opposed to neighboring or unrelated synapses, as shown in Fig. 2(a). In contrast, heterosynaptic plasticity, shown in Fig. 2(b), represents the ability of the synapse to modify its strength as a result of activity in another neighboring neuron or pathway [[40], [41], [42]]. The homosynaptic and heterosynaptic plasticity can be categorized in two types: potentiation or facilitation and depression, based on synaptic modification of the synapse. Moreover, depending on the durability of synaptic modification, plasticity is categorized into two types: short-term plasticity (short-term facilitation (STF) and short-term depression (STD)) and long-term plasticity (long-term potentiation (LTP) and long-term depression (LTD)). STF occurs when the repeatedly stimulated presynaptic neuron releases neurotransmitters at a faster rate, which causes a temporary enhancement in the strength of a synapse between neurons [37]. However, STD induces with the depletion of neurotransmitters and their vesicles despite the presence of presynaptic stimulations, thus weakens the strength of a synapse. Both STF and STD occur over a shorter period of time, lasting only tens of milliseconds to a couple of minutes [37,43]. LTP is a type of activity-dependent plasticity that persistently strengthens a synapse based on recent patterns of synaptic activity, thus producing a long-lasting increase in signal transmission between two neurons. LTD persistently weakens specific synapses in order to build a productive use of synaptic strengthening caused by LTP, and stabilizes neuronal circuit to facilitate encoding of new information [37,44]. Both LTP and LTD last hours or longer based on the patterns of stimulation. Homosynaptic STF, STD, LTP, and LTD are input specified which means that if a synapse has these plasticity traits, it doesn't spread to other synapses and only propagates synaptic transmission between two succeeding neurons [45]. In contrast, heterosynaptic LTP and LTD are associative. Because weak stimulation of a single pathway alone is not enough to induce LTP, continuous stimulation of another pathway can induce LTP in both pathways [46]. Heterosynaptic LTD weakens a synapse independently, despite the activity of the pre- or post-synaptic neurons, as a result of a modulatory interneuron firing to stabilize the synaptic transmission [37,44]. Heterosynaptic LTD (which might occur along with homosynaptic LTP) has the ability to sustain synaptic competition as well as balancing plasticity modifications [42]. Therefore, long-term and short-term plasticity are considered as one of the major neuromorphic foundation of learning and memory in neuronal communication as memories are thought to be encoded by synaptic modification [41,44].

Homosynaptic depression weakens the strength of synapse more rapidly than heterosynaptic depression, and the homosynaptic reuptake process occurs after a synapse exhibits LTP or STF. In homosynaptic depression, the rapid depletion of neurotransmitter vesicles in the presynaptic terminal causes synapses to weaken swiftly. This is due to the direct response to a synapse caused by repeated presynaptic activation, which causes a quicker onset of depression [47,48]. In contrast, heterosynaptic depression tends to be mediated by diffusible factors or modulators that act indirectly on the synaptic strengths of the multiple synapses and, therefore, operates on a slower timescale [49,50]. The reuptake process after LTP or STF is slower because it involves more complicated cellular processes, such as recycling vesicles, the re-synthesis of neurotransmitters, and membrane trafficking. This makes the weakening of the synapses slower than homosynaptic depression [47,51]. Additionally, homosynaptic depression causes the synapse to deplete essential resources (like neurotransmitters or calcium ions) more quickly, which in turn accelerates fatigue [52]. In contrast, heterosynaptic depression indirectly deplete resources at the affected synapse and homosynaptic reuptake and recovery are mediated by transporter proteins, vesicle reformation, and reloading of neurotransmitters, which take time and further slowdown the synaptic weakening process [53].

Heterosynaptic plasticity modifies the synaptic strengths between adjacent synapses depending on the overall network activity and contributes to the homeostatic regulation. When a synapse exhibits long-term potentiation (LTP), neighboring synapses experience long-term depression (LTD) to prevent over-excitation and subsequent neuron excitotoxicity [53,54]. However, in the case of modular input-specific roles, heterosynaptic plasticity facilitates synaptic competition by allowing increased activity or allocating specific pathways to reinforce (i.e., strengthening the synapse) the active synapse while inhibiting less active ones. This synaptic pruning of synapses refines connectivity and facilitates selective and effective information processing, which is especially important during development and adaptive learning [55,56].

The interaction between homo- and hetero- synaptic plasticity emphasizes the complexity of synaptic modification. Heterosynaptic plasticity enables the modulation of multiple synaptic pathways, in conjunction with highly active synapses (homosynaptic plasticity), thereby simplifying the information storage process of the brain. Consequently, this leads to an increase in brain connections, which in turn increases the complexity of network interactions necessary for memory encoding, association, and storage. In addition, during associative learning, activating two different pathways simultaneously (due to homosynaptic and heterosynaptic connections) can lead to the potentiation of one synapse while also altering the surrounding synaptic environment. Heterosynaptic changes are important for associative learning because they assist in embedding information in distributed networks and form an association between a conditioned stimulus and an unconditioned stimulus [57,58].

Neurons produce electrical pulses, namely action potentials, when activated individually (initiating homosynaptic plasticity) or collectively (initiating heterosynaptic plasticity) in synaptic transmission. These synchronized, rhythmic, or repetitive electrical pulses that result from mass or individual neural communication are called brainwaves. The CNS recognizes five kinds of brainwaves with different frequency bands: delta (δ), theta (θ), alpha (α), beta (β), and gamma (γ) [59]. Each brainwave, shown in Table 2, contributes to different neural activities (known as brain states) through synaptic modification. The mammalian hippocampal has a neutral frequency of 10 Hz, at which repetitive stimulation does not modify synaptic efficacy. However, higher-frequency bands of brainwaves induce synaptic modification for memory and learning [60]. Therefore, the brainwaves and their corresponding brain-states for regular and plasticity-dependent synaptic transmission are different. For instance, gamma brainwaves, or the coupling of theta and gamma brainwaves, facilitate the timing and synchronization of neuronal firing, thereby enhancing the efficacy of synaptic transmission and promoting long-term plasticity [61]. However, identifying the exact brainwaves responsible for specific types of synaptic plasticity is difficult and challenging due to the complexity of interacting brain rhythms [62], task and region specificity of brainwaves and plasticity [63], state-dependent brain activity [64], diverse plasticity mechanisms [65], technical limitations in measurement tools [66], and plasticity beyond synapses [67]. Therefore, we used delta, theta, and alpha (f ≤ 10 Hz) brainwaves to show the normal synaptic response and beta and gamma brainwaves to show the plasticity (both homo- and hetero- synaptic) responses of the proposed synapse.

**Table 2:** Characteristics and Frequency Bands of Brainwaves.

Band	Range	Brain-states
Delta (δ)	< 4 Hz	Sleep / Loss of bodily awareness
Theta (θ)	4 – 8 Hz	Inner peace / Meditation / Healing
Alpha (α)	8 – 12 Hz	Passive attention / Learning
Beta (β)	12 – 35 Hz	Anxiety dominant / Active / Learning
Gamma (γ)	> 35 Hz	Concentration / Awareness / Intelligence

## Architecture of neuromemristive synapse

**Fig. 3:**
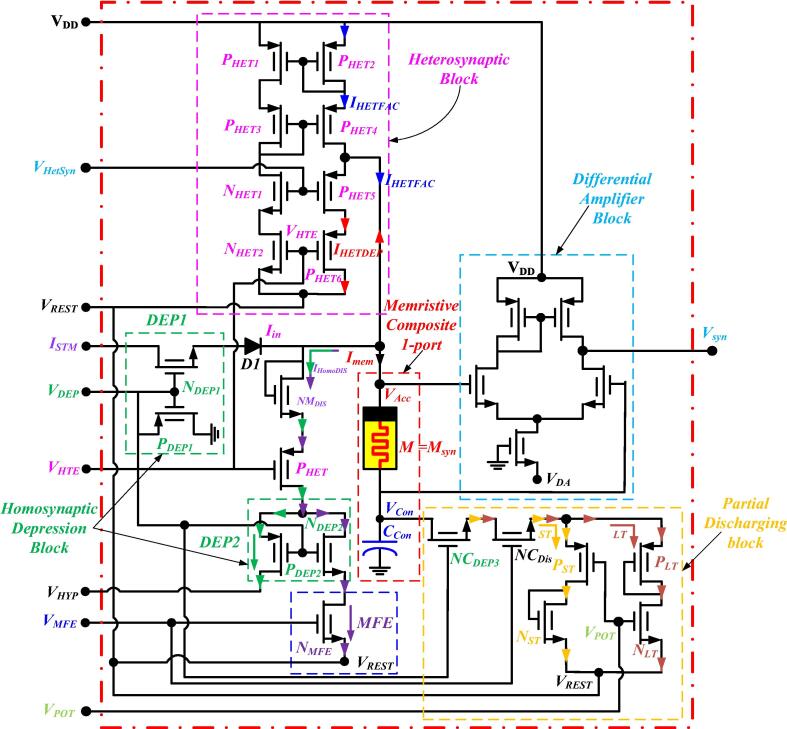
Circuit diagram of the proposed neuromemristive synapse. The design comprises several functional blocks: a memristive composite 1-port that mimics the bio-realistic attributes of a synapse and its plasticity; a homosynaptic depression block for short-term and long-term depression (STD/LTD); a partial discharging block for short-term and long-term potentiation (STF/LTP); a heterosynaptic block for heterosynaptic facilitation and depression; and an amplifier block that produces the final synaptic output, V_Syn_.

**Fig. A1:**
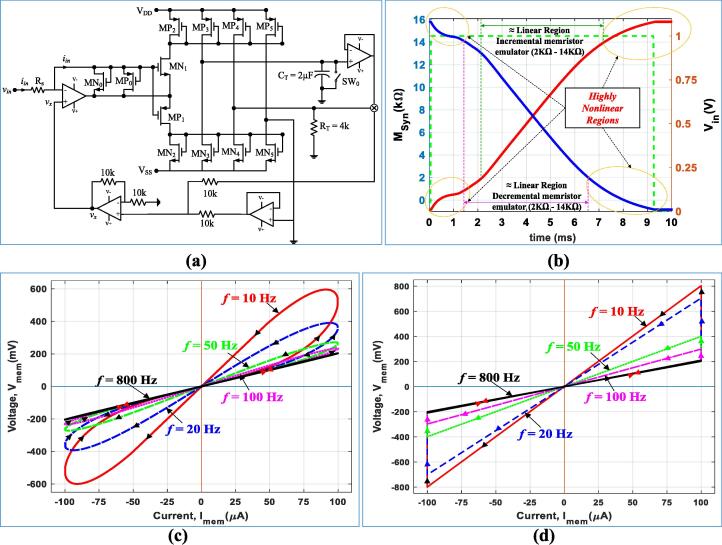
(a) Circuit diagram of the incremental memristor emulator that used to design the neuro-memristive synapse. (b) Memristance vs. time relationship of the incremental and decremental memristor emulator. Frequency-dependent pinched hysteresis loops for zero-mean periodic stimulation of ***I_in_ =* 100 µ*A*** for (**a**) bi-polar sinusoidal input, and (**b**) pulse input with frequencies ***f = 10 Hz, 20 Hz, 50 Hz, 100 Hz, and 800 Hz***.

The proposed neuromemristive synapse, shown in Fig. 3, contains several blocks. A composite 1-port that consists of a memristor (M) and a capacitor (C_CON_) in series is the main core of the neuromemristive synapse. The composite 1-port has a current-controlled push–pull architecture where the memristor is used to mimic the bio-realistic attributes of a synapse and its plasticity, whereas the capacitor is used to control the ratio of discharging through memristor M. In the active cycle of homosynaptic (I_mem_ = I_in_ = I_STM_) or heterosynaptic (I_mem_ = I_HTEFAC_) stimulation, both memristor M and capacitor C_CON_ will be charged by I_mem_ current (*i.e.*, the devices are pulling I_mem_ to charge themselves) and in the inactive cycle of I_mem_ = I_in_ = I_STM_ or I_mem_ = I_HTEFAC_ (shown in blue arrowheads in heterosynaptic block), the stored charge in C_CON_ will be forced to discharge either partially or fully through memristor M (*i.e.*, C_CON_ is pushing M to discharge through homosynaptic (I_HomoDIS_, shown in purple arrowheads) or heterosynaptic (I_HTEDEP,_ shown in red arrowheads in heterosynaptic block) discharge pathways based on synaptic activity and their corresponding plasticity phenomena. The I_mem_ can be defined as,

(1) $$I_{\mathrm{mem}}=\left\{\begin{array}{c} I_{\mathrm{in}}=I_{\mathrm{stm}},V_{\mathrm{DEP}}>0\&\&V_{\mathrm{HTE}}>0\&\&V_{\mathrm{HteSyn}}<0\\ I_{\mathrm{HTEFAC}},V_{\mathrm{DEP}}>0\&\&V_{\mathrm{HTE}}>0\&\&V_{\mathrm{HteSyn}}>0\\ \begin{array}{c} I_{\mathrm{HomoDIS}}=\frac{V_{\mathrm{Con}}-V_{\mathrm{Acc}}}{M},\left(V_{\mathrm{DEP}}<0orV_{\mathrm{MFE}}>0\right)\&\&V_{\mathrm{HTE}}<0\&\&V_{\mathrm{HteSyn}}>0\\ I_{\mathrm{HTEDEP}}=\frac{V_{\mathrm{Con}}-V_{\mathrm{Acc}}}{M},V_{\mathrm{DEP}}=0\&\&V_{\mathrm{MFE}}=0\&\&V_{\mathrm{HTE}}<0\&\&V_{\mathrm{HteSyn}}<0 \end{array} \end{array}\right)$$

where V_Acc_ is the accumulated voltage of the memristive composite 1-port, V_Con_ is the voltage of the control capacitor, and M = M_Syn_ is the memristance of the artificial neuromemtistive synapse. The memristance (M_Syn_) can be define as

(2) $$M=M_{\mathrm{Syn}}=M\left(0\right)+V_{C_{T}}R_{T}$$

where, M(0) is the initial state and, $V_{C_{T}}$ ($V_{C_{T}}$ depends on inputs) and *R_T_* are intrinsic parameters of the memristor as shown in Fig. A1 in the Appendix. The diode *D_1_* in Fig. 3 is used to block the reverse directional current. The artificial synaptic voltage (V_Syn_) is equal to the voltage across the memristor (V_mem_) and is extracted using a differential amplifier as shown in Fig. 3 where,

(3) $$V_{\mathrm{Syn}}=V_{\mathrm{mem}}=I_{\mathrm{mem}}M=V_{\mathrm{Acc}}-V_{\mathrm{Con}}$$

To implement the homosynaptic potentiation (STF and LTP) and depression (STD and LTD) and reuptake (*i.e.*, equivalent to MFE in electronic circuit), we implement separate discharging paths through memristor M and capacitor C_CON_. According to section 3, the pattern, activity, durability, and the ratio of the decrement in synaptic efficacy of a synapse for the MFE and homosynaptic STD or LTD are quite different. Therefore, we designed two separate discharging paths to implement the MFE and homosynaptic STD or LTD phenomena. The composite 1-port will be discharged through HOMO_MFE pathway (C_CON_ → M → NM_DIS_ → P_HET_ → N_DEP2_ → N_MFE_ → V_REST_) for MFE, as shown with purple arrowheads in Fig. 3. In contrast, the composite 1-port will be discharged through HOMO_DEP pathway (C_CON_ → M → NM_DIS_ → P_HET_ → P_DEP2_ → V_HYP_) for homosynaptic STD or LTD, as shown with green arrowheads in Fig. 3. V_REST_ and V_HYP_ represent the resting membrane potential (−70 mV) and hyperpolarize membrane potential (−110 mV), respectively. Moreover, the accumulation of synaptic efficacy and the rate of abolishment of neurotransmitters from synaptic cleft for homosynaptic STF and LTP are quite different according to section 2. Therefore, we implement two separate discharge paths through C_CON_ capacitor. When the composite 1-port exhibits homosynaptic LTP, the stored charge in C_CON_ will mostly discharged through HOMO_LT_PART_DIS pathway (C_CON_ → NC_DEP3_ → NC_DIS_ → P_LT_ → N_LT_ → V_REST_), as shown with burgundy arrowheads, whereas it will marginally discharge through HOMO_MFE pathway (C_CON_ → M → NM_DIS_ → P_HET_ → N_DEP2_ → N_MFE_ → V_REST_), as shown with purple arrowheads in Fig. 3. In contrast, the stored charge in C_CON_ will mostly discharged through memristor M using HOMO_MFE pathway (C_CON_ → M → NM_DIS_ → P_HET_ → N_DEP2_ → N_MFE_ → V_REST_), as shown with purple arrowheads, and partially discharge through HOMO_ST_PART_DIS pathway (C_CON_ → NC_DEP3_ → NC_DIS_ → P_ST_ → N_ST_ → V_REST_), as shown with yellow arrowheads in Fig. 3, for homosynaptic STF. We implemented additional depression switches DEP1 (N_DEP1_ and N_DEP2_) and NC_DEP3_, MFE switch (N_MFE_), C_Con_ partial discharging switch (NC_Dis_), and heterosynaptic switch (P_HET_) to ensure that the neuromemristive synapse shouldn’t exhibit two different types of plasticity phenomena at the same time similar to biological synapses.

The heterosynaptic facilitation and depression are implemented using the heterosynaptic block, shown in Fig. 3, which are connected to the memristor M of the composite 1-port. When the neuromemristive synapse exhibits heterosynaptic facilitation (*i.e.*, active cycle of V_HTE_ where V_HTE_ = V_H_), the hetero-synapse input V_HetSyn_ (V_HetSyn_ = V_H_) generates I_HETFAC_ current (where, I_mem_ = I_HETFAC_) and charges the memristor M and capacitor C_CON_ through HET_FAC pathway (V_DD_ → P_HET2_ → P_HET4_ → M → C_CON_ → 0). In contrast, for heterosynaptic depression (*i.e.*, inactive cycle of V_HTE_ where V_HTE_ = V_L_ and V_HetSyn_ = V_L_), memristor M and capacitor C_CON_ are discharged through HET_DEP pathway (0 → C_CON_ → M → P_HET5_ → P_HET6_ → V_REST_).

## Results and discussions

A modified memristor emulator [68] was used in this paper to design the proposed neuromemristive synapse as the memristor is commercially unavailable. We operated the memristor emulator in the linear region (2 K ∼ 14 K) to avoid the unintended consequences of nonlinear ionic dopant drift at its boundaries [69,70] as shown in Fig. A1(b) in the appendix. The memristive fingerprints of the modified memristor emulator (M(0) = 2 K, C_T_ = 2µF, and R_T_ = 4 K), shown in Fig. A1 (a), for bipolar inputs are shown in Figs. A1 (c) and (d). We conducted various SPICE simulations to verify the bio-realistic features of the proposed neuromemristive synapse. Table 4 lists the circuit parameters used to obtain the subsequent experimental results of the proposed synapse, using the operating signals mentioned in Table 3.

**Table 3:** Operating modes of the proposed neuromemristor synapse.

Synapse Type	Memory Type	Synaptic Mode	Synaptic Response	Operating Signals				
				V_HTE_	V_HteSyn_	V_DEP_	V_MFE_	V_POT_
Homosynaptic	Volatile	Facilitation/ Potentiation	NSR + MFE	V_L_	V_0_	V_H_	V_H_	V_0_
			STF + MFE	V_L_	V_0_	V_H_	V_H_	V_L_
			LTP + MFE	V_L_	V_0_	V_H_	V_H_	V_H_
		Depression	STD	V_L_	V_0_	V_L_	V_0_	V_L_
			LTD	V_L_	V_0_	V_L_	V_0_	V_H_
	Nonvolatile	Facilitation/ Potentiation	NSR	V_L_	V_0_	V_H_	V_0_	V_0_
			STF	V_L_	V_0_	V_H_	V_0_	V_0_
			LTP	V_L_	V_0_	V_H_	V_0_	V_0_

Heterosynaptic	Volatile	Facilitation	HTE_FAC	V_H_	V_H_	V_H_	V_0_	V_0_
		Depression	HTE_DEP	V_L_	V_L_	V_H_	V_0_	V_0_

**Table 4:** Circuit parameters of the proposed neuromemristive synapse.

**Parameters**	**Value**	**Parameters**	**Value**
V_DD_	2.5 V	V_SS_	−2.5 V
V_H_	0.4 ∼ 1.0 V	V_L_	−1.0 ∼ -0.4 V
V_REST_	−70 mV	V_HYP_	−110 mV
V_0_	0 V	C_Con_	12 µF

### Non-plastic synaptic responses

**Fig. 4:**
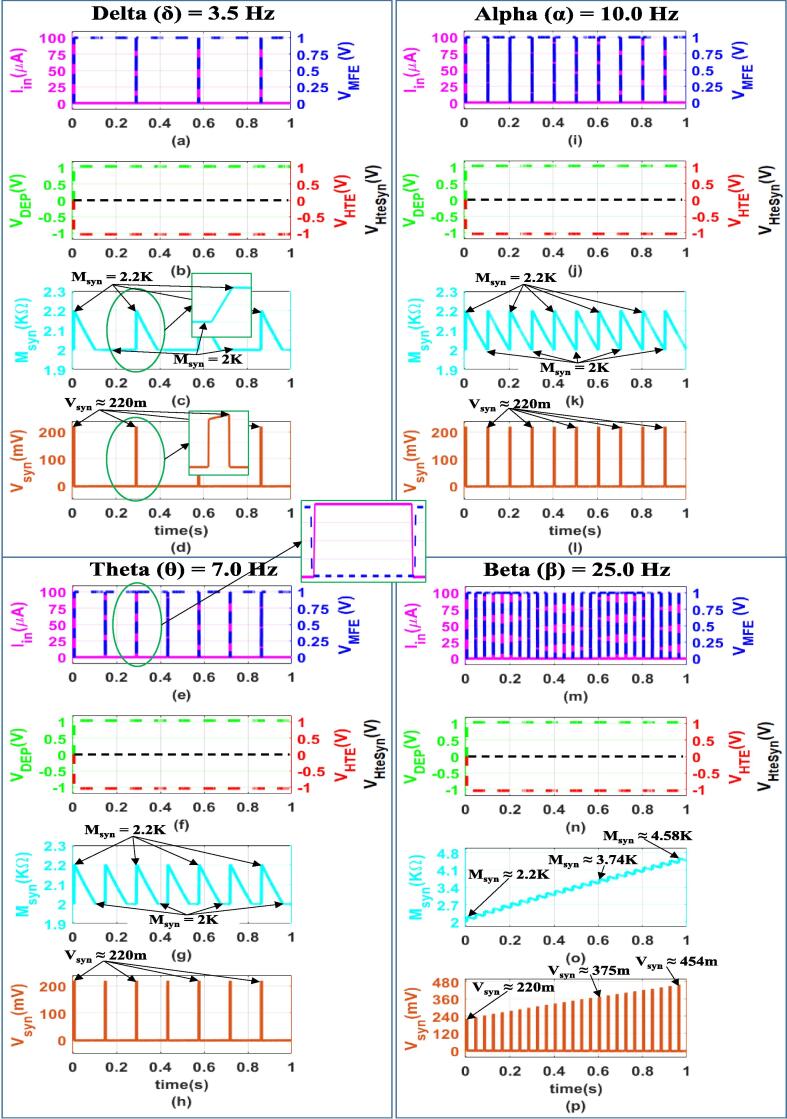
Synaptic response of the proposed neuromemristive synapse for Delta (δ), Theta (θ), Alpha (α) and Beta (β) brainwaves. Input stimulation (**I_in_**) and memory fading effect (**V_MFE_**) for (a) *δ* = 3.5 Hz, (e) θ = 7 Hz, (i) α = 10 Hz, and (m) β = 25 Hz. Control signals for homosynaptic depression (**V_DEP_**) and heterosynaptic plasticity (**V_HTE_**) for (b) *δ* = 3.5 Hz, (f) θ = 7 Hz, (j) α = 10 Hz, and (n) β = 25 Hz. Rate of change of the synaptic strength (**M_syn_**) for (c) *δ* = 3.5 Hz, (g) θ = 7 Hz, (k) α = 10 Hz, and (o) β = 25 Hz. Artificial synapse voltage (**V_syn_**) for (d) *δ* = 3.5 Hz, (h) θ = 7 Hz, (l) α = 10 Hz, and (p) β = 25 Hz.

We used a stimulating pulse current I_in_ = I_stm_ = 100 µA with PW = 1 ms along with brainwave frequencies *δ* = 3.5 Hz (PP = 286 ms), θ = 7 Hz (PP = 142.86 ms), and α = 10 Hz (PP = 100 ms), which can be seen in magenta in Fig. 4(a), (e), and (i). This resulted in the proposed memristive synapse responding in a manner that does not exhibit synaptic plasticity. In contrast, we used the same I_in_ = 100 µA (PW = 1 ms and PP = 40 ms) and β-brainwaves (β = 25 Hz) to show the response of our proposed architecture to plasticity, as shown in Fig. 4(m). Fig. 4, Fig. 4, Fig. 4, Fig. 4 show the 1′s complement MFE signals (V_MFE_ = V_H_ = 1 V) in blue. The inset in the middle shows that V_MFE_ is inactive when I_in_ is active and vice versa. The proposed synapse does not exhibit either homosynaptic depression or heterosynaptic plasticity, as evidenced by the values of V_DEP_ = V_H_ = 1 V, V_HTE_ = V_L_ = −1V, and V_HteSyn_ = V_0_ = 0 V, as shown in Fig. 4 (b), (f), (j), and (n). The artificial synapse doesn't build up synaptic strength (M_syn_) for *δ* = 3.5 Hz, θ = 7 Hz, and α = 10 Hz brainwaves (Fig. 4, Fig. 4, Fig. 4. However, M_syn_ builds up significantly for β = 25 Hz (Fig. 4(o)). Like the biosynapse, the neuromemristive synapse doesn't change the strength of the synapse below the neutral frequency (≤ 10 Hz); instead, it changes the efficacy of the synapse above the neutral frequency (> 10 Hz). The artificial synaptic voltage (V_syn_) consequently increases in each active cycle of I_in_, as shown in Fig. 4(p). This is because the synaptic strength (M_syn_) builds up at a non-neutral frequency of β = 25 Hz. This is in contrast to Fig. 4, Fig. 4, Fig. 4, where V_syn_ remains the same (V_syn_ = 220 mV) due to constant M_syn_ at *δ* = 3.5 Hz, θ = 7 Hz, and α = 10 Hz. We can conclude from Fig. 4 that the proposed artificial synapse qualitatively mimics the biorealistic attributes of a biosynapse.

### Homosynaptic plasticity

**Fig. 5:**
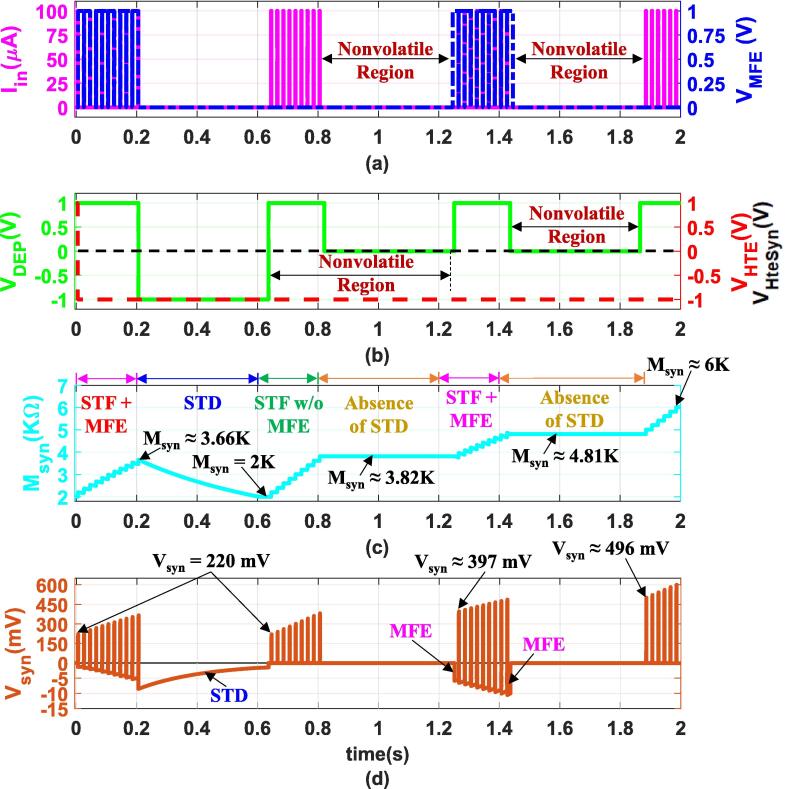
Short-term plasticity response of the proposed neuromemristive synapse for Gamma-brainwave (50 Hz). (a) Input stimulus (***I_in_***) and memory fading effect (***V_MFE_***), (b) control signals for homosynaptic depression (***V_DEP_***) and heterosynaptic plasticity (***V_HTE_***), (c) rate of change of the synaptic strength (***M_syn_***), and (d) synaptic voltage (***V_syn_***) of the proposed artificial synapse.

**Fig. 6:**
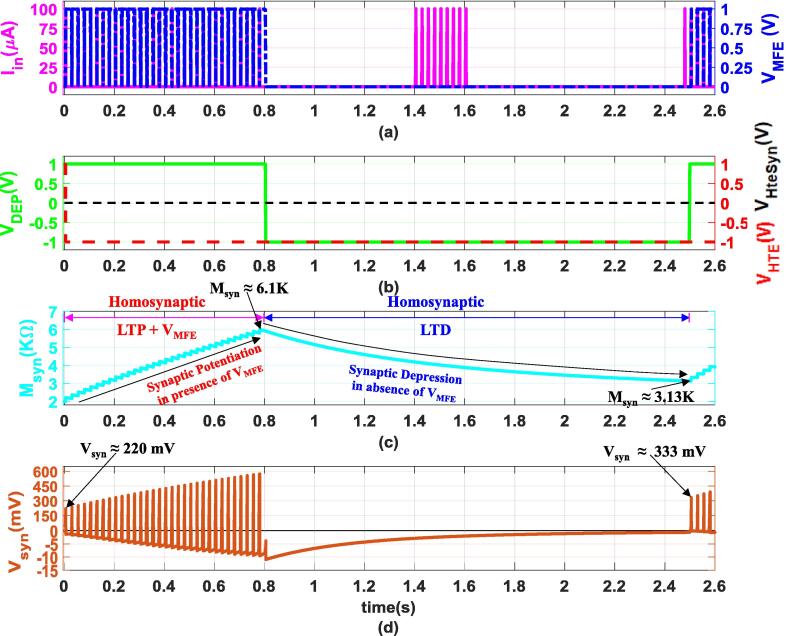
Long-term plasticity response of the proposed neuromemristive synapse for Gamma-brainwave (40 Hz). (a) Input stimulus (***I_in_***) and memory fading effect (***V_MFE_***), (b) control signals for homosynaptic depression (***V_DEP_***) and heterosynaptic plasticity (***V_HTE_***), (c) rate of change of the synaptic strength (***M_syn_***), and (d) synaptic voltage (***V_Syn_***) of the proposed artificial synapse.

In this paper, we implemented both homosynaptic short-term and long-term plasticity, as shown in Fig. 5, Fig. 6, respectively. We showed short-term plasticity by stimulating the proposed neuromemristive synapse with a range of different conditions, including short-term facilitation (STF), short-term depression (STD), and non-volatility stimuli. On the contrary, for long-term plasticity, we provided long-term potentiation (LTP) and long-term depression (LTD) stimuli.

To demonstrate the short-term plasticity of the proposed synapse, stimuli were applied using homosynaptic spike trains with specific parameters. An input STF stimuli I_in_ = 100 µA (PW = 1 ms) with γ-brainwaves (γ = 50 Hz (PP = 20 ms)) was used along with active memory fading effect signal V_MFE_ on the first and third cycles, as shown in Fig. 5(a). Fig. 5(b) shows the homosynaptic STD (V_DEP_) and the inactive heterosynaptic plasticity (V_HTE_ = V_L_ = -1V and V_HteSyn_ = V_0_ = 0 V) signals. It can be seen in Fig. 5(c) that the close successive stimulus gradually raises the synaptic efficacy (M_syn_), even when V_MFE_ is active (1st cycle, t ≤ 0.2 s). In contrast, the artificial synapse gradually decreases its synaptic efficacy in the active period of STD (0.2 s < t < 0.64 s) with a similar passion to that of the volatile biosynapse. However, a non-volatility increase in M_syn_ is seen in the active cycle of I_in_ and the inactive cycle of V_MFE_ (0.64 s ≤ t < 0.84 s), and M_syn_ remains unchanged (M_syn_ = 3.82 K) until V_DEP_ ≥ 0 and V_MFE_ = 0 (0.84 s ≤ t ≤ 1.24 s). Similar like the biological synapse, the synaptic efficacy (M_syn_) of the proposed neuromemristive synapse decreases in the inactive cycle of I_in_ and active cycle of memory fading effect (V_MFE_) along with the active cycle of homosynaptic STD (V_DEP_). In contrast, the proposed synapse increases its synaptic efficacy (M_syn_) in the active cycle of I_in_ despite the presence of V_MFE_ and in absence of V_DEP_. Fig. 5 (c) also shows that when the input stimulus I_in_ returns, the synaptic efficacy (M_syn_) rises in three stages: namely, a volatile rise (1.24 s < t ≤ 1.46 s), a hold-off rise (1.46 s < t ≤ 1.8 s), and a nonvolatile rise (t > 1.8 s). Synaptic voltage (V_syn_), shown in Fig. 5(d), produces a higher voltage (t ≥ 1.2 s) compared to t ≤ 0.84 s due to higher buildup in M_syn_.

To determine the outcome of the long-term plasticity of the artificial synapse, we stimulated the proposed synapse with homosynaptic LTP stimuli I_in_ = 100 µA (PW = 1 ms) and **γ**-brainwaves (**γ =** 40 Hz **(**PP = 25 ms)) shown in magenta solid, and the V_MFE_ is shown in dotted blue in Fig. 6(a). Fig. 6(b) shows the homosynaptic LTD (V_DEP_) and the inactive heterosynaptic plasticity (V_HTE_ = V_L_ = −1V and V_HteSyn_ = V_0_ = 0 V) in solid green and dotted red and black curves, respectively. Fig. 6(c) shows that the synaptic efficacy (M_syn_) swiftly increases in the potentiation cycle despite the presence of active V_MFE_ and gradually decreases in the depression cycle (active presence of V_DEP_) despite of the active presence of I_in_ (1.4 s ≤ t ≤ 1.62 s). The memristive synapse can't go back to its initial state M(0) = 2 K during the LTD period because synaptic efficacy has already grown during the LTP period. Hence, the proposed synapse produces higher V_syn_ (shown in Fig. 6(d)) when the presynaptic stimulations return to the active state after t ≥ 2.4 s than that in the initial period (t < 0.1 s).

We can conclude from Fig. 6 that the neuromemristive synapse effectively imitates the long-term plasticity phenomenon of the biosynapse, as long-lasting enhancements in synaptic efficacy were developed in both the LTP and LTD processes, similar to that of biological synapses.

### Heterosynaptic plasticity

**Fig. 7:**
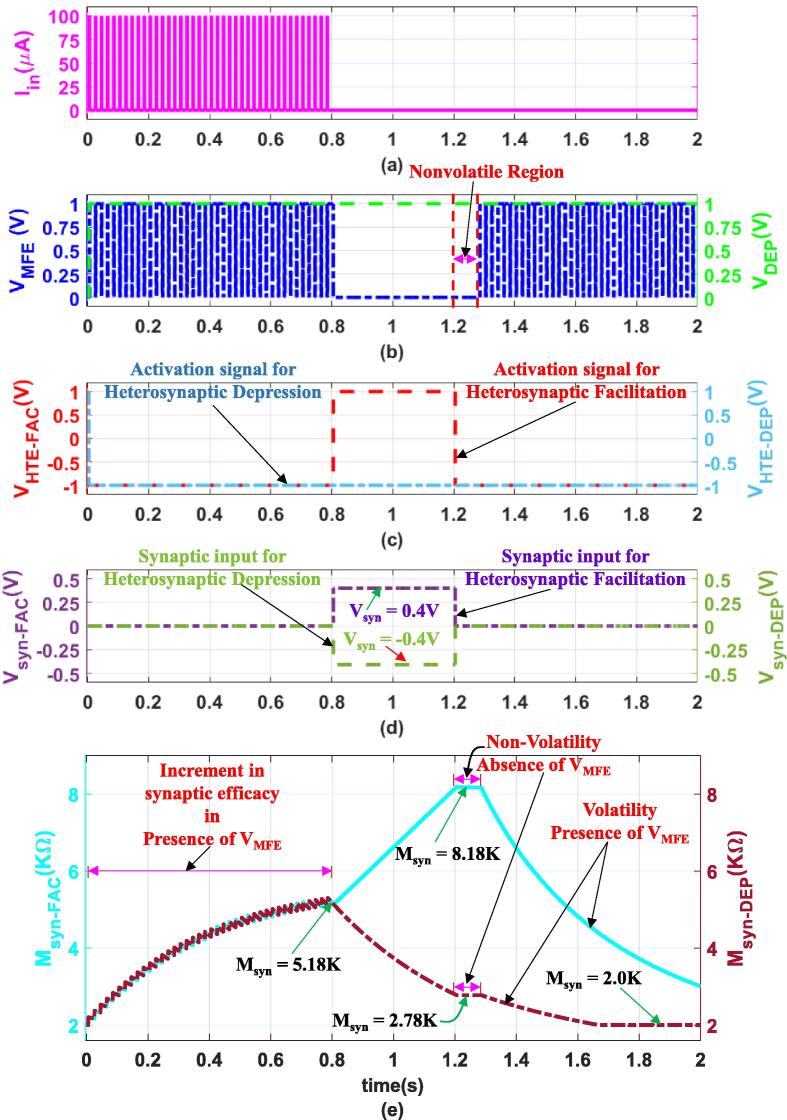
Heterosynaptic plasticity response of the proposed neuromemristive synapse for Gamma-brainwave (50 Hz). (a) Input stimuli (***I_in_***), (b) homosynaptic ***V_MFE_*** and ***V_DEP_***, (c) control signals for heterosynaptic facilitation (***V_HTE-FAC_***) and depression (***V_HTE-DEP_***), (d) synaptic inputs for heterosynaptic facilitation (***V_syn-FAC_***) and depression (***V_syn-DEP_***), and (e) rate of change in synaptic strength for facilitation (***M_syn-FAC_***) and depression (***M_syn-DEP_***).

To implement the heterosynaptic plasticity, we provide a homosynaptic stimuli I_in_ = 100 µA (PW = 1 ms) with **γ**-brainwaves (**γ =** 50 Hz **(**PP = 20 ms)) and active duty cycle of 40 %, shown in Fig. 7(a), to the proposed synapse. The homosynaptic stimuli was provide for the readers' better understanding. Fig. 7(b) shows the 1-s complement V_MFE_ and V_DEP_. The control signals for heterosynaptic facilitation (V_HTE-FAC_) and depression (V_HTE-DEP_) are shown in Fig. 7(c). Fig. 7(d) shows the synaptic input voltages for heterosynaptic facilitation (V_syn-FAC_) and depression (V_syn-DEP_). Combination of these synaptic inputs and control signals are responsible for the generation of heterosynaptic facilitation and depression in the proposed neuromemristive synapse. We provide only 40 % of V_H_ = |V_L_| = 1 V as synaptic inputs for facilitation (V_syn-FAC_ = 400 mV) and depression (V_syn-DEP_ = -400 mV) to maintain the bio-realistic phenomenon that the pathways for homosynaptic plasticity strengthen the synapse profoundly (due to the higher number of neurotransmitters released in those specific pathways) than that of heterosynaptic plasticity. Fig. 7(e) shows the rate of the changes in synaptic strengths for both homo and heterosynaptic facilitation (M_syn-FAC_) and depression (M_syn-DEP_) inputs. Observe that both the M_syn-FAC_ and M_syn-DEP_ are increasing monotonically despite the presence of active V_MFE_ over the period of 0 s ≤ t < 0.8 s. When heterosynaptic facilitation occurs at the neuromemristive synapse, the synaptic strength (M_syn-FAC_) builds up swiftly due to V_syn-FAC_. On the other hand, when heterosynaptic depression happens at the neuromemorristive synapse, the synaptic strength (M_syn-DEP_) slowly decreases over the active period of 0.8 s to 1.2 s because of V_syn-DEP_. Fig. 7 shows that both the M_syn-FAC_ and M_syn-DEP_ keep their synaptic strengths (nonvolatile operation) from 1.2 s to 1.3 s, but they both start to lose strength when the active cycle of V_MFE_ starts up again after 1.4 s. Observe from Fig. 7(e) that the M_syn-DEP_ returns to the initial value M_syn-DEP_ (0) = 2 K and holds the initial states despite the active presence of V_MFE_.

**Fig. 8:**
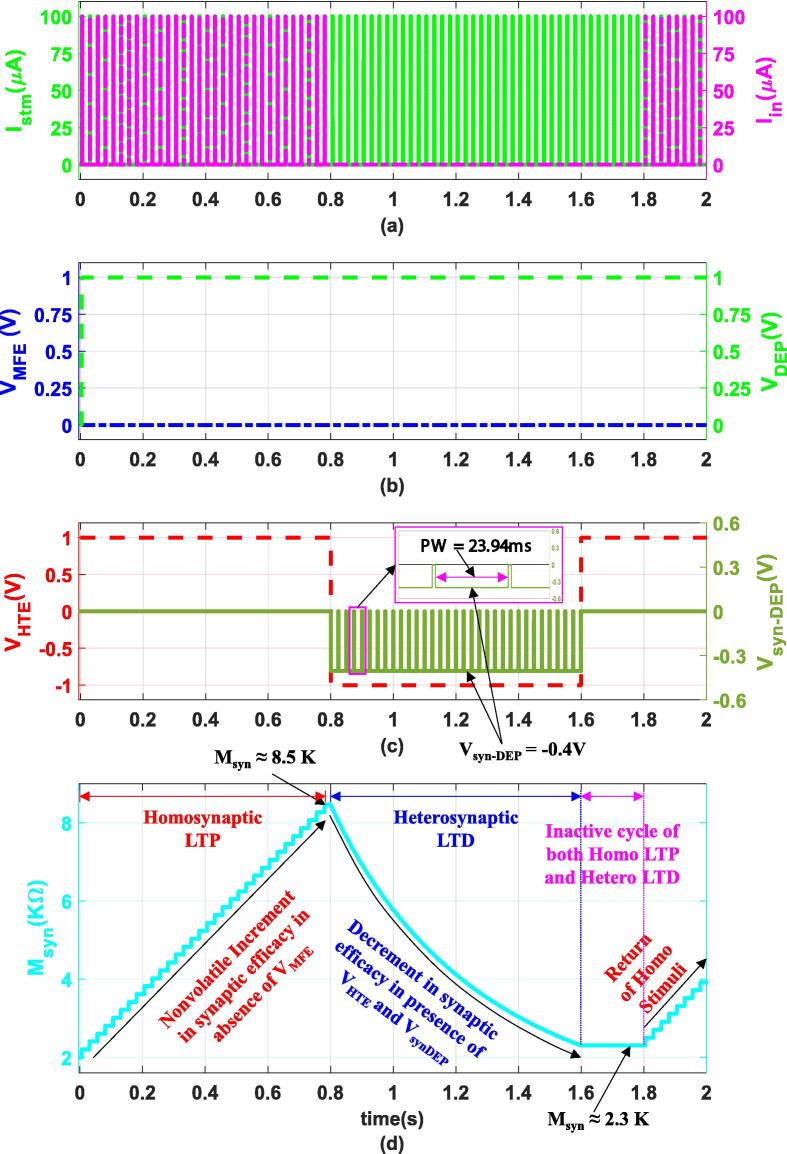
Response of the proposed neuromemristive synapse on the homeostatic role of the heterosynaptic plasticity for Gamma-brainwave (50 Hz). (a) External stimulations (**I_stm_**) and input stimuli (**I_in_**), (b) homosynaptic **V_MFE_** and **V_DEP_**, (c) heterosynaptic control signals (**V_HTE_**) and synaptic inputs for heterosynaptic depression (**V_syn-DEP_**), (d) rate of change in synaptic strength (**M_syn_**).

The homeostatic role of heterosynaptic plasticity (i.e., homeostasis) is that when one synapse experiences long-term potentiation (LTP), neighboring synapses go through long-term depression (LTD) to prevent over-excitation or excitotoxicity. To demonstrate homeostasis, the neuromorphic synapse was potentiated over t < 0.8 s and t > 1.8 s via homosynaptic LTP stimuli. We then depress the proposed synapse over 0.8 s ≤ t ≤ 1.6 s using heterosynaptic LTD stimuli. For homosynaptic LTP, we provide I_stm_ = 100 µA (PW = 1 ms) with **γ**-brainwaves (**γ =** 40 Hz **(**PP = 25 ms)) as shown in green in Fig. 8(a). We employed the nonvolatile mode of the neuromemristive synapse to perform homosynaptic potentiation by setting V_MFE_ = 0 V and V_DEP_ = V_H_ = 1 V, as shown in Fig. 8(b). Fig. 8(c) shows that the the control signal V_HTE_ = V_L_ = −1V and synaptic input voltage V_syn-DEP_ = −400 mV are facilitating heterosynaptic LTD over a time span 0.8 s ≤ t ≤ 1.6 s. Observe from magenta I_in_ in Fig. 8(a) that the memristive synapse only exhibiting input stimuli I_in_ = I_stm_ for V_HTE_ = V_H_ = 1 V over the time period t < 0.8 s and t ≥ 1.8 s. The synaptic efficacy (M_syn_), shown in Fig. 8(d), of the proposed synapse is monotonically increasing up until it reaches its highest point: M_syn_ ≈ 8.5 K for the active homosynaptic sequential I_in_ stimuli over t ≤ 0.8 s and t ≤ 1.8 s. On the other hand, M_syn_ is gradually decreasing and approaching the initial state Msyn ≈ 2.3 K due to active heterosynaptic LTD generated by V_HTE_ = V_L_ = −1V and V_syn-DEP_ = −400 mV for 0.8 s ≤ t ≤ 1.6 s. Fig. 8 indicates that the proposed neuromemristive synapse is capable of performing both homosynaptic LTP and heterosynaptic LTD when given the appropriate control and stimulating signals. It also proves that the neuromemristive synapse can maintain homeostasis (i.e., homeostatic regulation) by demonstrating heterosynaptic LTD, while the nearby artificial synapse may experience homosynaptic LTP. Such occurrences are necessary for maintaining neural or neuronal network stability and preventing excitotoxicity [53,54].

**Fig. 9:**
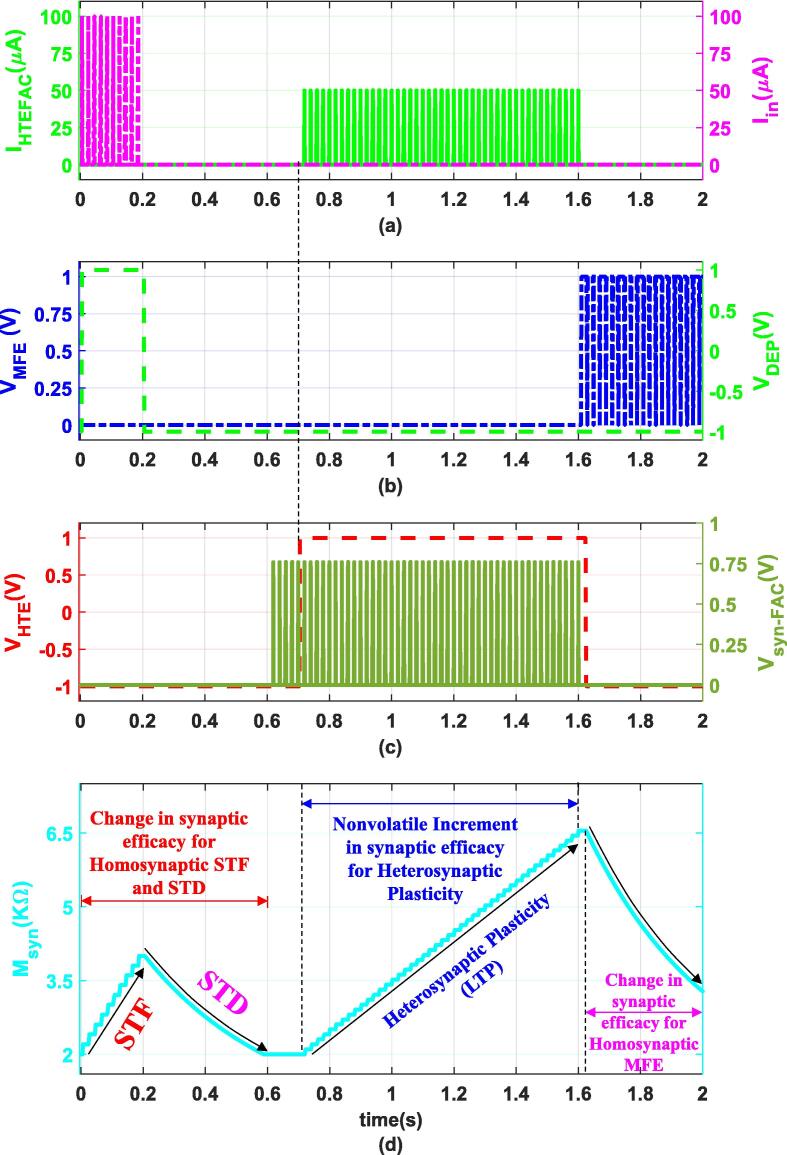
Modular input-specific plasticity response of the heterosynaptic plasticity of the proposed neuromemristive synapse for Gamma-brainwave (50 Hz). (a) Input stimuli (***I_in_***) and heterosynaptic facilitation stimuli (***I_HTEFAC_***), (b) homosynaptic ***V_MFE_*** and ***V_DEP_***, (c) heterosynaptic control signals (***V_HTE_***) and synaptic inputs for heterosynaptic facilitation (***V_syn-FAC_***), (d) rate of change in synaptic strength (***M_syn_***).

Modular input-specific role of heterosynaptic plasticity facilitates synaptic competition by allowing increased activity or allocating specific pathways to strengthen the synapse. In order to illustrate the modular input-specificity of heterosynaptic plasticity, we first applied homosynaptic short-term plasticity (STF and STD) to the neuromorphic synapse over a time span of 0 s to 0.6 s. For t < 0.2 s, we give homosynaptic STF stimuli I_in_ = 100 µA (PW = 1 ms) along with γ-brainwaves (γ = 50 Hz (PP = 20 ms)), which can be seen in magenta in Fig. 9(a). We used the neuromemristive synapse in a nonvolatile configuration for homosynaptic STF (V_MFE_ = 0 over t ≤ 1.6 s) and depressed it with V_DEP_ = −1V for homosynaptic STD over 0.2 s ≤ t < 2 s, as shown in Fig. 9(b). From Fig. 9(a), it can be seen that the neuromemristive synapse is being stimulated by heterosynaptic LTP stimuli I_HTEFAC_ = 50 µA (PW = 1 ms) with γ = 50 Hz (PP = 20 ms) over 0.7 s ≤ t < 1.6 s. These stimuli are generated by V_syn-FAC_ = V_H_ = 0.75 V (PW = 1 ms) with γ = 50 Hz (PP = 20 ms) over 0.6 s ≤ t < 1.6 s , as shown in Fig. 9(c). The heterosynaptic control signal V_HTE_ = V_H_ = 1 V over 0.7 s ≤ t ≤ 1.65 s and for the rest of the time span V_HTE_ = V_H_ = -1V, as shown in Fig. 9(c). Observe from Fig. 9, Fig. 9 that the proposed synapse generates I_HTEFAC_ (green in Fig. 9(a)) only while V_HTE_ = V_H_ = 1 V (red in Fig. 9(c)). Fig. 9(d) shows that the artificial synaptic strength (M_syn_) is swiftly increasing for both homosynaptic STF (t < 0.2 s) and heterosynaptic LTP (0.7 s − t ≤ 1.65 s). On the other hand, it is steadily decreasing for homosynaptic STD (0.2 s ≤ t ≤ 0.6 s) and gradually decreases for homosynaptic MFE (t ≥ 1.65 s). Fig. 9 shows that we can modulate the potentiation or depression activity of the specific pathways to strengthen or weaken the proposed neuromemristive synapse. It also proves that the proposed synapse qualitatively imitates the synaptic pruning mechanism of bio-synapses as it refines the connectivity of the specific pathways and facilitates selective and effective information processing.

**Fig. 10:**
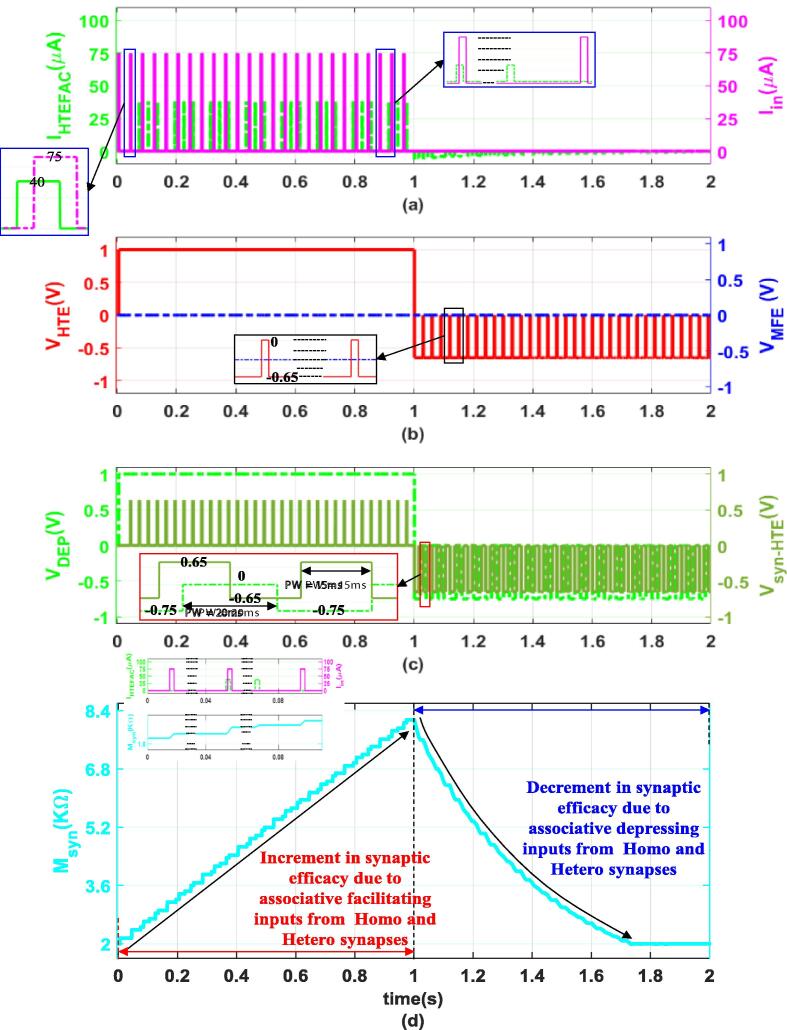
Response of the proposed neuromemristive synapse for associative role of the heterosynaptic plasticity for Beta-brainwave (25 Hz for Homosynaptic and 33 Hz for Heterosynaptic synapse). (a) External stimulations (**I_stm_**) and input stimuli (**I_in_**), (b) homosynaptic **V_MFE_** and heterosynaptic control signals (**V_HTE_**) (c) homosynaptic depression signal (**V_DEP_**) and synaptic inputs for heterosynaptic synapse (**V_syn-HTE_**), (d) rate of change in synaptic strength (**M_syn_**).

To implement the associative role of the heterosynaptic plasticity, we provide a homosynaptic stimuli I_in_ = 75 µA (PW = 1 ms) with β = 25 Hz (PP = 20 ms) and a heterosynaptic stimuli I_HTEFAC_ = 40 µA (PW = 1 ms) with β = 33 Hz (PP = 30.3 ms) as shown in Fig. 10(a). For this homosynaptic simulation, we didn’t consider the memory fading effect (i.e., V_MFE_ = 0 V) as we want the neuromemristive synapse to be operated in nonvolatile mode. A bipolar signal was provided as a heterosynaptic plasticity controlling signal with V_HTE_ = V_H_ = 1 V for t < 1 s and V_HTE_ = V_L_ = −0.65 V for t ≥ 1 s with a duty ratio of 49.5 % as shown in Fig. 10(b). Fig. 10(c) illustrates the homosynaptic depression (V_DEP_) and heterosynaptic synaptic signal (V_syn-HTE_) during both facilitation (t < 1) and depression (t ≥ 1) cycles. Fig. 10(d) shows that the synaptic efficacy (M_syn_) of the neuromemristive synapse was potentiated over the time span t < 1 s due to the combinational homosynaptic (I_in_ > 0) and heterosynaptic (I_HTEFAC_ > 0) facilitation stimuli where V_DEP_ > 0, V_HTE_ > 0, and V_syn-HTE_ > 0. However, the synaptic efficacy (M_syn_) neuromemristive synapse was depressed within 1 s ≤ t ≤ 2 s due to the combinational homosynaptic (V_DEP_ < 0) and heterosynaptic (V_syn-HTE_ < 0) depression stimuli where V_HTE_ < 0. It is interesting to note that both homo and hetero synaptic facilitation stimuli helped M_syn_ build up during the potentiation phase, while both homo and hetero synaptic depression stimuli weakened M_syn_. Therefore, we can say that the suggested neuromemristive synapse can continue associative learning as it turns on both homosynaptic and heterosynaptic pathways at the same time and makes one synapse stronger. In contrast, during the depression phase, the system modifies the surrounding synaptic environment by depressing the neuromemristive synapse, while another neuromemristive synapse undergoes long-term projection (LTP) within a neuronal or neural network.

**Fig. 11:**
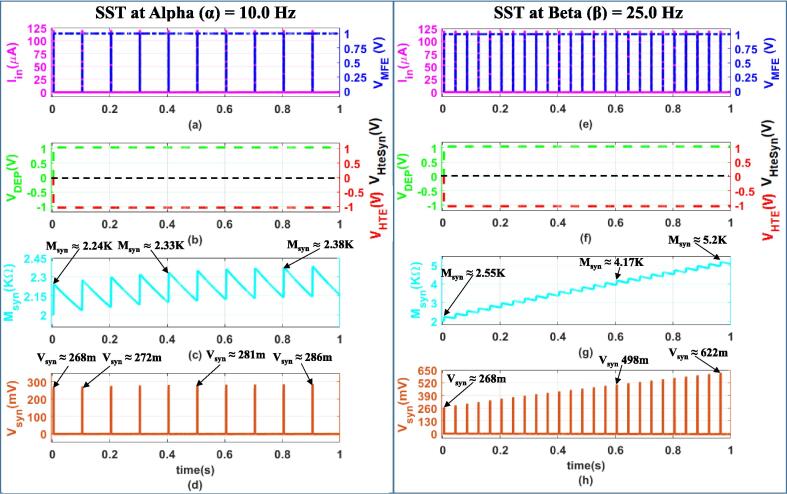
Strong stimulation response of the proposed neuromemristive synapse. Strong stimulation stimuli ***I_in_*** and 1′s complimentary memory fading effect signal ***V_MFE_*** signals (a) for Alpha-brainwaves (10 Hz) and (e) for Beta-brainwaves (25 Hz). Control signals for homosynaptic depression and heterosynaptic mode operations (b) for Alpha-brainwaves (10 Hz) and (f) for Beta-brainwaves (25 Hz). Rate of change of the synaptic strength (***M_Syn_***) (c) for Alpha-brainwaves (10 Hz) and (g) for Beta-brainwaves (25 Hz). Synaptic voltage (***V_Syn_***) of the proposed artificial synapse (d) for Alpha-brainwaves (10 Hz) and (h) for Beta-brainwaves (25 Hz).

### Strong stimulation

**Fig. 12:**
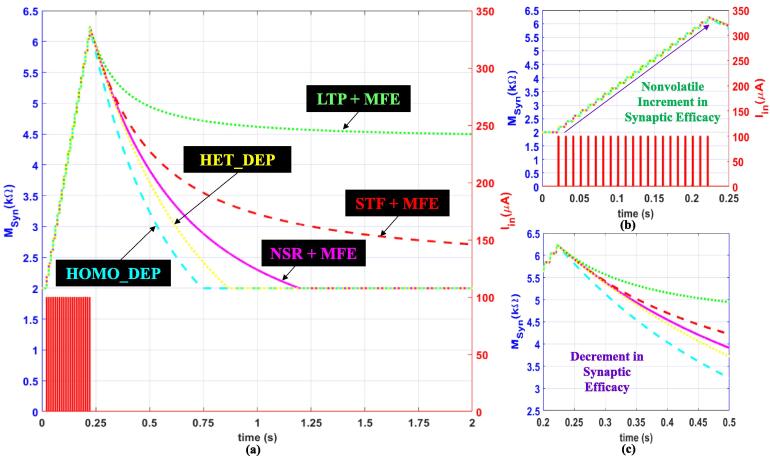
Comparison of the synaptic weakening (M_syn_) of proposed neuromemristive synapse for different plasticity modes. Observe that the synaptic efficacy of the proposed neuromemristive synapse weakens faster for homosynaptic depression than heterosynaptic depression and other synaptic operations, which act similarly to the biological synapse.

To investigate the response of the proposed neuromemristive synapse to strong stimulating phenomena, the same amplitude of I_in_ = 120µA (PW = 1 ms) at two different brainwaves, α = 10 Hz (PP = 100 ms) and β = 25 Hz (PP = 40 ms) was provided, as shown in Figs. 11 (a) and (e) , respectively. Observe that the stimulating inputs in Fig. 4, Fig. 12, as well as Fig. 4, Fig. 12, are indifferent, except for the pulse amplitude. V_MFE_ signals in Fig. 4, Fig. 12, as well as Fig. 4, Fig. 12, remain the same. Figs. 11(b) and (f) show the homosynaptic LTD (V_DEP_) and the inactive heterosynaptic plasticity (V_HTE_ = V_L_ = −1V and V_HteSyn_ = V_0_ = 0 V) signals. Fig. 11(c) shows that the neuromemristive synapse (M_Syn_) builds up its efficacy at neutral frequency (α = 10 Hz) for strong stimulation, whereas it did not modify its efficacy for regular stimulation in Fig. 4(k). Observe from Fig. 11(g) that the neuromemristive synapse accumulates higher synaptic strength (M_Syn_) for strong stimuli than in Fig. 4(o) at β = 25 Hz. Figs. 11(d) and (h) show a higher production of synaptic voltage (V_syn_) compared to Fig. 4, Fig. 4. Strong stimulation enhances the synaptic strength of the artificial synapse more than regular stimulation, thereby increasing the likelihood of postsynaptic firings. Therefore, we can conclude that the neuro-memristive synapse effectively imitates the strong stimulation of the biosynapse.

### Comparison of synaptic weakening process for different plasticity modes

Section 2 emphasizes notable disparities in the synaptic weakening rate of a specific biosynapse during homosynaptic depression and homosynaptic reuptake, in contrast to heterosynaptic depression. The rate of synaptic weakening over time is dependent upon prior stimulations, similar to LTP, STF, or NSR. A biosynapse exhibiting LTP requires significantly more time to weaken its efficacy than that of STF and NSR exhibiting synapses. Therefore, to demonstrate the synaptic weakening attributes of the proposed neuromemristive synapse, the same input stimuli (I_in_ = 100µA, PW = 1 ms, and PP = 10 ms (**γ = 1**00 Hz)) was provided to the different synaptic modes, as shown in Fig. 12. The neuromemristive synapse monotonically increases its synaptic efficacy (M_syn_) in the active cycle of I_in_ for the synaptic modes of homosynaptic NSR, STF, and LTP, homosynaptic depression (STD or LTD), and heterosynaptic depression, as shown in Fig. 12(b). Observe that the nonvolatile increment of the M_syn_ is exactly the same (0 s ≤ t ≤ 0.22 s) for all the aforementioned synaptic modes. The proposed synapse initiates the synaptic weakening process at the same time (t > 0.22 s), as shown in Fig. 12(c). The homosynaptic NSR, STF, and LTP-exhibiting synapses initiate weakening only through the active MFE route, and both homosynaptic and heterosynaptic depressions weaken through their specified routes. Fig. 12 shows that the artificial synapse acts like the biological one and swiftly weakens its efficacy in homosynaptic depression compared to heterosynaptic depression. Moreover, it weakens faster than that of NSR, STF, and LTP exhibiting synapse with active V_MFE_. Similar to biosynapse, the synaptic strength of the proposed LTP exhibiting artificial synapse decreases more gradually than that of STF and NSR. Therefore, we can conclude that the synaptic weakening response of the proposed neuromemristive synapse is qualitatively equivalent to the attributes of a biological synapse.

### Power dissipation of the proposed neuromemristive synapse

Table 5 illustrates the maximum power dissipation per cycle of the proposed neuromemristive synapse and the synapses presented in [21] and [22] across several synaptic operation modes**.** The neuromemristive synapse exhibits the highest power consumption in the heterosynaptic modular input-specific function, whereas the homosynaptic non-plastic synaptic response (NSR) consumes the least power. Factors such as the amplitude, frequency, and pulse width of both homosynaptic and heterosynaptic stimulations significantly influence the power dissipation. The proposed synapse demonstrates reduced power dissipation during long-term plasticity and strong stimulation (β = 25 Hz) compared to the power dissipation reported in [21] and [22] under similar input conditions as shown in Table 5. Table 5 also shows that the proposed synapse exhibits significantly higher power dissipation during short-term plasticity. Such higher power dissipation occurs due to variations in homosynaptic stimulation patterns compared to those in [21] and [22]. The power consumption of the proposed synapse is qualitatively comparable to that of biological synapses, which require substantial energy to process information for homosynaptic long-term synaptic plasticity, strong stimulations, and heterosynaptic plasticity functions.

**Table 5:** Power dissipation of the proposed neuromemristive, ref [21], and ref [22] synapses in a cycle for different synaptic modes of operations.

Synapse Type	Synaptic Response	Power Dissipations of Proposed Neuromemristive Synapse (µW)	Power Dissipations of Ref [21] Synapse (µW)	Power Dissipations of Ref [22] Synapse (µW)
Homosynaptic	Nonplastic Synaptic Response (NSR)	113.4	283.673	196.553
	Synaptic Response for β = 25 Hz	189.15	−	
	Short-term Plasticity (STF + STD)	280.612	137.85 ( STF+MFE)	131.994
	Long-term Plasticity (LTP + LTD)	444.659	694.43 ( LTP+MFE)	452.95
	Strong Stimulation (SST)	416.574 (I_in_ = 120µA)	643.244 (I_in_ = 130µA)	424.921 (I_in_ = 130.2 µA)

Heterosynaptic	Heterosynaptic Facilitation	465.59	−	−
	Heterosynaptic Depression	409.005	−	−
	Homeostasis role	451.325	−	−
	Modular Input-specific role	670.555	−	−
	Associative role	642.585	−	−

### Synaptic performance, limitations, and future perspectives

The proposed neuromemristive synapse exhibits robustness due to its bio-realistic operation, energy efficiency, and CMOS compatibility, which helps overcome the mechanical instability and stochastic switching of alternative technologies. The proposed neuromemristive synapse demonstrates robustness due to its bio-realistic operation, energy efficiency, and CMOS compatibility. These attributes allow it to overcome the mechanical instability and stochastic switching prevalent in alternative technologies mentioned in the introduction section. Compared to other state-of-the-art existing synapses, our proposed synapse demonstrates high specificity. It mimics distinct biological mechanisms, including both homo- and heterosynaptic plasticity (STF, STD, LTP, LTD), reuptake processes, and input-specific associativity properly, which other synapses lack. We utilize a single memristor and a controlled capacitor to design the proposed synapse, which highlights the scalability due to the inherent miniaturization potential of memristive devices, their low power consumption, and their area-efficient architecture. In addition, the proposed synapse allows for post-fabrication calibration. The calibration can be achieved by initializing the memristor within a linear region (2 K–14 K), adjusting transistor bias, and controlling the system input. Specifically, we can control the resting membrane (V_REST_) and hyperpolarize membrane (V_HYP_) potentials to calibrate the transistors, as these voltages control the discharge rate for the memory fading effect, homosynaptic depression, heterosynaptic depression, and partial discharging blocks. Therefore, the proposed synapse might be suitable for large-scale integration in spiking neural networks (SNNs) and a viable platform for brain-like intelligence.

Despite the aforementioned promising results, one of the limitations of this study is the absence of physical fabrication data, since we confined the validation to simulation environments. SPICE simulations provide critical insights into functional behavior; however, they are unable to illustrate the consequences of process variation and reliability issues that are inherent in physical hardware. Moreover, we present a successful operation of a single synapse without system-level integration, which is insufficient to guarantee performance in a large-scale neuromorphic system. Therefore, our proposed synapse still needs to prove its path to large-scale manufacturing and integration into functional neuromorphic computing systems. Our near future work will therefore focus on fabricating the synapse in a standard CMOS process and characterizing its performance, variability, and endurance. The subsequent objective is to demonstrate the operation of small-scale spiking neural networks (SNNs) with Hebbian, anti-Hebbian, and associative learning using these neuromemristive synapses and neurons to validate their collective learning capabilities and scalability. Additionally, we want to fabricate an industrial design of the proposed neuromemristive synapse and utilize it in designing human brain-like intelligence in future.

## Conclusion

The proposed neuromemristive synapse mimics the bio-realistic characteristics of a synapse and its plasticity according to the brainwaves of various brain states. It imitates the homosynaptic short- and long- term plasticity (STF, STD, LTP, and LTP) as well as the LTP, LTD, homeostatic, and modular input-specific roles of heterosynaptic plasticity. The proposed synapse also exhibits reuptake and diffusion mechanisms, which involve the removal of neurotransmitters from the synaptic cleft. Furthermore, the proposed synapse demonstrates bio-realistic behaviors of a neuron, such as strong stimulation, an exponentially decaying conductance trace, and voltage-dependent synaptic responses. Various SPICE simulations verified the mimicking features of the proposed artificial synapse in relation to biological phenomena.

Unlike multiple memory elements-based prior artificial synapses, the proposed neuromorphic circuit was implemented in a circuit with off-the-shelf devices and can be utilized in both volatile and nonvolatile configurations. It is not simply another synaptic model, relying solely on mathematical or macro-models of the memristor. We can implement the proposed power- and area- efficient bio-inspired synapse in miniature CMOS ICs because the mem-conductive neuromorphic architectures overcome the limitations of graphene, liquid crystal carbon nanotube composites, subthreshold analog- and transistor-based CMOS neural circuits, and conventional Von-Neumann architectures. In addition, academics can also utilize it to analyze the synaptic functionalities of neurons, replacing the need for in vivo or in vitro analysis.

## Data availability

We will make data from simulations and circuit implementations available upon request.

## Declaration of generative AI in scientific writing

We wrote and revised the manuscript by ourselves. We used QuillBot Premium only as a grammatical checker. We accepted some of QuillBot’s suggestions for clarity in the sentences.

## CRediT authorship contribution statement

**Zubaer Ibna Mannan:** Conceptualization, Formal analysis, Investigation, Methodology, Project administration, Resources, Software, Supervision, Validation, Visualization, Writing – original draft, Writing – review & editing. **Sami Azam:** Formal analysis, Funding acquisition, Investigation, Project administration, Resources, Supervision, Validation, Visualization, Writing – review & editing. **Ram Kaji Budhathoki:** Formal analysis, Investigation, Resources, Validation, Visualization, Writing – review & editing. **Nur Alam:** Formal analysis, Investigation, Resources, Validation, Visualization, Writing – review & editing. **Hyongsuk Kim:** Funding acquisition, Investigation, Project administration, Resources, Supervision, Visualization, Writing – review & editing.

## Declaration of competing interest

The authors declare that they have no known competing financial interests or personal relationships that could have appeared to influence the work reported in this paper.

## Appendix

A modified memristor emulator [68], illustrated in Fig. A1(a), was employed to design the proposed neuromemristive synapse due to the unavailability of commercial memristors. The emulator was operated within the linear resistance range (2 kΩ –14 kΩ) to mitigate undesired effects stemming from nonlinear ionic dopant drift at boundary conditions [69,70], as illustrated in Fig. A1(b). The memristive fingerprints [71] of zero-crossing and frequency-dependent pinched hysteresis loops of the modified memristor emulator (M(0) = 2 K, C_T_ = 2µF, and R_T_ = 4 K) for bipolar sinusoidal and pulse waves are shown in Figs. A1 (c) and (d), respectively. The pinched hysteresis loop lobe area decreases with increasing frequency, converging to a single-valued straight line for *f* ≥ 100  Hz. This behavior confirms that the emulator exhibits the defining characteristics of a memristor [71].

## Footnotes

1 Synaptic cleft, shown in Fig. 1(b), is a small gap between pre- and post- synaptic neurons (≈ 20 nm wide) and forms a junction between neurons.
